# Proposed Memorial to Dr. Ranking

**Published:** 1912-11-23

**Authors:** J. J. Webb

**Affiliations:** Secretary. General Hospital, Tunbridge Wells


### The Editor's Letter-Box.

### Proposed Memorial to Dr. Ranking.

*To the Editor of* The Hospital.

Sir,—It is hoped that you will be good enough to insert the following particulars respecting a proposed memorial to Dr. Ranking. An influential committee has been formed to promote a fund, which is to be devoted to commemorate the eminent services, to both rich and poor, of the late Dr. John E. Ranking, who was killed in a motor-car accident recently.

For over thirty years he rendered most devoted and most successful services to the General Hospital at Tunbridge Wells, and it is proposed to apply the fund in some practical way for the benefit of the sick and suffering poor. The form which the memorial will take naturally depends upon the amount forthcoming. It is hoped that the sum will be a substantial one, so as to admit of some form of permanent endowment. Cheques may be sent to Mr. F. Wadham Elers, chairman, General Hospital, Tunbridge Wells; Mrs. Stone-Wigg, 32 Molyneux Park, Tunbridge Wells; Mr. Evelyn Pardington, Glynlee, Tunbridge Wells; the Rev. H. Masters, M.A., Speldhurst Rectory; and Mr. J. S. Snelgrove, Kingswood, Tunbridge Wells.—Yours faithfully,

J. J. Webb, Secretary.

General Hospital, Tunbridge Wells,
November 12.

